# DOF AFFECTING GERMINATION 2 is a positive regulator of light-mediated seed germination and is repressed by DOF AFFECTING GERMINATION 1

**DOI:** 10.1186/s12870-015-0453-1

**Published:** 2015-03-04

**Authors:** Silvia Santopolo, Alessandra Boccaccini, Riccardo Lorrai, Veronica Ruta, Davide Capauto, Emanuele Minutello, Giovanna Serino, Paolo Costantino, Paola Vittorioso

**Affiliations:** Dipartimento di Biologia e Biotecnologie “C. Darwin”, Sapienza Università di Roma, Piazzale Aldo Moro 5, 00185 Rome, Italy; Istituto Pasteur Fondazione Cenci Bolognetti, Dipartimento di Biologia e Biotecnologie “C. Darwin”, Sapienza Università di Roma, Piazzale Aldo Moro 5, 00185 Rome, Italy

**Keywords:** DAG2, Seed germination, DAG1, *Arabidopsis thaliana*

## Abstract

**Background:**

The transcription factor DOF AFFECTING GERMINATION1 (DAG1) is a repressor of the light-mediated seed germination process. DAG1 acts downstream PHYTOCHROME INTERACTING FACTOR3-LIKE 5 (PIL5), the master repressor, and negatively regulates gibberellin biosynthesis by directly repressing the biosynthetic gene *AtGA3ox1*. The Dof protein DOF AFFECTING GERMINATION (DAG2) shares a high degree of aminoacidic identity with DAG1. While *DAG1* inactivation considerably increases the germination capability of seeds, the *dag2* mutant has seeds with a germination potential substantially lower than the wild-type, indicating that these factors may play opposite roles in seed germination.

**Results:**

We show here that *DAG2* expression is positively regulated by environmental factors triggering germination, whereas its expression is repressed by PIL5 and DAG1; by Chromatin Immuno Precipitation (ChIP) analysis we prove that DAG1 directly regulates *DAG2*. In addition, we show that Red light significantly reduces germination of *dag2* mutant seeds.

**Conclusions:**

In agreement with the seed germination phenotype of the *dag2* mutant previously published, the present data prove that DAG2 is a positive regulator of the light-mediated seed germination process, and particularly reveal that this protein plays its main role downstream of PIL5 and DAG1 in the phytochrome B (phyB)-mediated pathway.

**Electronic supplementary material:**

The online version of this article (doi:10.1186/s12870-015-0453-1) contains supplementary material, which is available to authorized users.

## Background

The DNA BINDING WITH ONE FINGER (Dof) proteins are a family of plant-specific transcription factors characterised by a single zinc-finger DNA-binding domain. So far Dof proteins have been identified in *Chlamydomonas reinharditii,* where only one *Dof* gene is present, in ferns, mosses and in higher plants [1-3].

The number of *Dof* genes varies depending on the species; bioinformatic analysis of the *Arabidopsis* and rice genome predicts 36 and 30 *Dof* genes, respectively [1], while 26 are present in barley [2], 31 in wheat [4], and 28 in sorghum [5]. Members of this family have been found to be involved in the regulation of diverse plant-specific processes. Although the biological role of many Dof proteins has not been clarified yet, a number of them has been shown to be involved in responses to light and phytohormones, as well as in seed development and germination [6-15].

Seed germination is regulated by environmental factors such as light, temperature and nutrients, and by phytohormones, particularly gibberellins (GA) and abscissic acid (ABA) [16]. The effect of light is mediated mainly by the photoreceptor phytochrome B (phyB) [17], and light modulates in opposite ways the levels of GA and ABA, as it induces GA biosynthesis and causes a reduction in ABA levels [18,19]. Among the factors involved in phyB-mediated GA-induced seed germination, the bHLH transcription factor PHYTOCHROME INTERACTING FACTOR 3-LIKE 5 (PIL5) represents the master repressor of this process in *Arabidopsis* [20].

We have previously shown that inactivation of the Dof proteins DAG1 and DAG2 affects in opposite ways seed germination: *dag2* mutant seeds required more light and GA than wild-type seeds to germinate, whereas germination of *dag1* seeds was less dependent on these factors [7,8,21].

Recently, we have also pointed out that DAG1 acts as a negative regulator in the phyB-mediated pathway: *DAG1* gene expression is reduced in seeds irradiated for 24 hours with Red light, and this reduction is dependent on PIL5; in *pil5* mutant seeds *DAG1* expression is reduced irrespective of light conditions, indicating that DAG1 acts downstream of PIL5. Moreover, DAG1 negatively regulates GA biosynthesis by directly repressing the GA biosynthetic gene *AtGA3ox1* [22]. Very recently we showed that in repressing *AtGA3ox1* DAG1 directly interacts with the GA INSENSITIVE (GAI) DELLA protein [23]. Furthermore, we pointed out that DAG1 plays a role also in embryo development, as inactivation of *DAG1* results in a significant number of embryo abnormalities [7,24], and simultaneous inactivation of both *DAG1* and *GAI* results in an embryo-lethal phenotype. Here, we provide evidence suggesting that DAG2, opposite to DAG1, functions as a positive regulator in the molecular pathway controlling seed germination, and that it is negatively regulated by DAG1.

Differently from DAG1, DAG2, although it is expressed during embryo development, is not likely to play a role in this process, as *dag2* mutant embryos develop similarly to wild-type embryos.

## Results

### *DAG2* inactivation affects phyB-dependent seed germination

We have previously demonstrated that *dag2* mutant seeds have a reduced germination potential, as they are substantially more dependent than the wild-type on the stimuli that promote germination [8]. This germination phenotype is opposite to that of *dag1* mutant seeds. As we have recently shown that DAG1 is a component of the phyB-mediated pathway controlling seed germination in *Arabidopsis* [22,23], we set up to verify whether DAG2 is also a component of this regulatory network.

**Figure 1:**
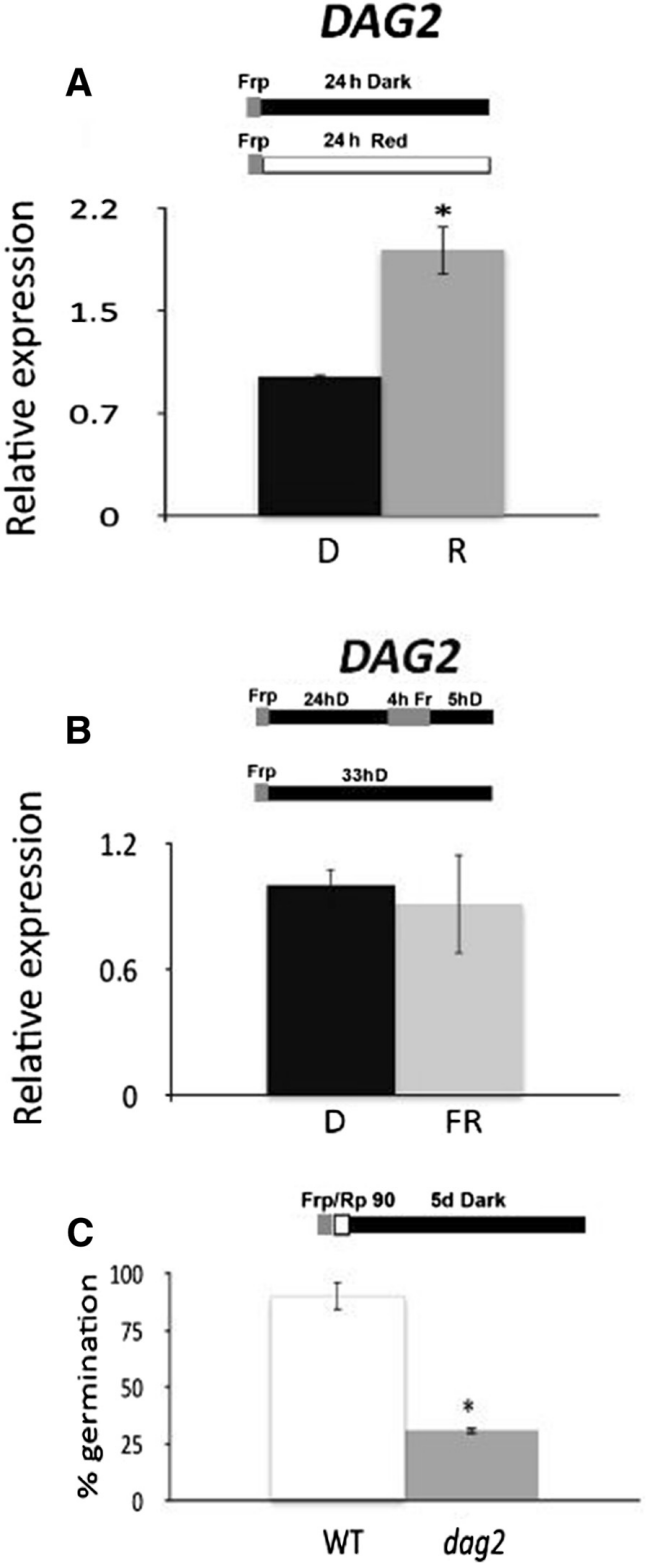
**Mutation of** ***DAG2*** **affects seed germination under R light.** Relative expression level of *DAG2* in wild-type seeds imbibed 24 hours in the dark (D), or under phyB-dependent conditions, **(A)**, and in the dark or under phyA-dependent conditions **(B)**. Relative expression levels were normalized with that of the *UBQ10* (*At4g05320*) gene, and are presented by the ratio of the corresponding mRNA level in Dark, which was set to 1. Similar results were obtained from three independent experiments, and a typical result is presented with SD values.Germination rates of wild-type and *dag2* mutant seeds, grown 5 days under phyB-dependent germination conditions **(C)**. Error bars = SEM. The diagram at top depicts the light treatment scheme for the experiment. FRp, Far Red pulse (40 μmol m^−2^ s^−1^); Rp, Red pulse (90 μmol m^−2^ s^−1^). Significative differences were analyzed by *t*-test (*P ≤ 0,05).

Since seed germination, although promoted mainly by phyB, may be induced also by phyA under very low light fluences [17], we checked whether Red (R) or Far Red (FR) light may control expression of the *DAG2* gene. Analysis of wild-type seeds exposed to phyB- or phyA-dependent conditions, according to Oh *et al*. 2006 [25], revealed that the *DAG2* gene is induced by exposure to R light (Figure 1A), whereas *DAG2* expression in seeds exposed to FR light was not significantly different than in seeds kept in the dark (Figure 1B). To assess whether DAG2 plays its role under R light, we analysed seed germination under phyB-dependent conditions [22] using the *dag2* mutant previously characterised [8], compared to the corresponding wild-type (Ws-4). Germination of *dag2* mutant seeds was significantly lower than that of wild-type seeds (30% and 90%, respectively - Figure 1C), thus confirming that DAG2 plays a positive role in seed germination and showing that it acts in the phyB-mediated pathway.

**Figure 2:**
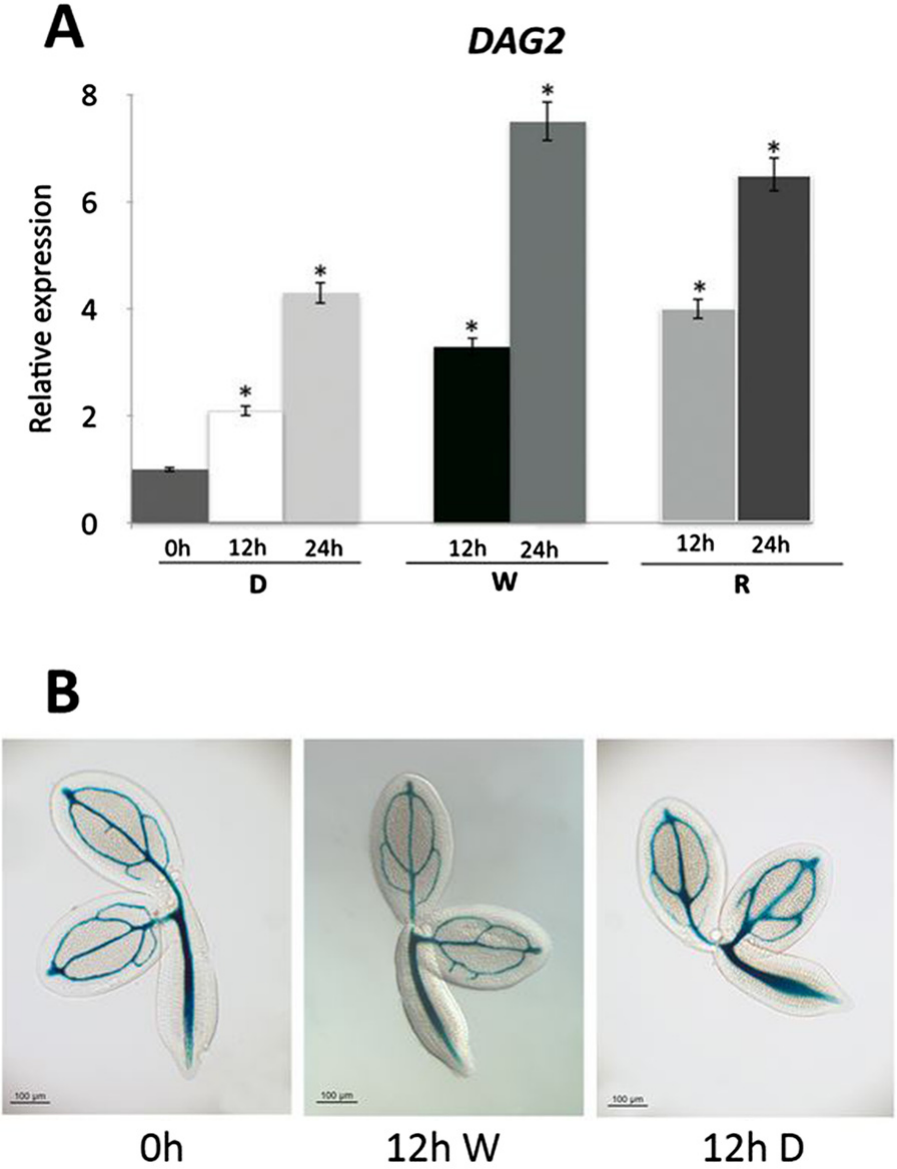
***DAG2*** **expression is induced by imbibition.** Relative expression level of *DAG2* in wild-type dry seeds (0 h), or imbibed 12 (12 h) or 24 hours (24 h) in the dark (D) or under White (W) or Red (R) light **(A)**. Relative expression levels were normalized with that of the *ACTIN2* (*At3g18780*) gene, and are presented by the ratio of the corresponding mRNA level in dry seeds, which was set to 1. Similar results were obtained from three independent experiments, and a typical result is presented with SD values. Significative differences were analyzed by *t*-test (*P ≤ 0,05). Histochemical staining of *DAG2:GUS* dry seeds, or imbibibed 12 hours under W light (W) or in the dark (D) **(B)**.

Since water uptake is a fundamental requirement for seed germination, we verified whether expression of *DAG2* was regulated during imbibition. We performed RT-qPCR assays on wild-type (Ws-4) dry seeds, and on seeds imbibed under White (W) and R light or in the dark for 12 and 24 hrs. Figure 2A shows that, compared to the low amount present in dry seeds, *DAG2* expression in seeds was much increased following water uptake in the dark (2 and 4 fold, respectively, at 12 and 24 hrs). Interestingly, the increase in *DAG2* mRNA level in seeds exposed to W or R light was even higher, probably due to the effect of both light and imbibition (3.7 and 7.8 fold in W light and 4 and 7-fold in R light, at 12 and 24 hrs, respectively - Figure 2A). GUS histological assays, performed on seeds of the *DAG2:GUS* transgenic line [8], dry or imbibed 12 hours under W light or in the dark respectively, showed that the *DAG2* promoter was active only in the vascular tissue (Figure 2B).

### *DAG2* is directly regulated by DAG1

**Figure 3:**
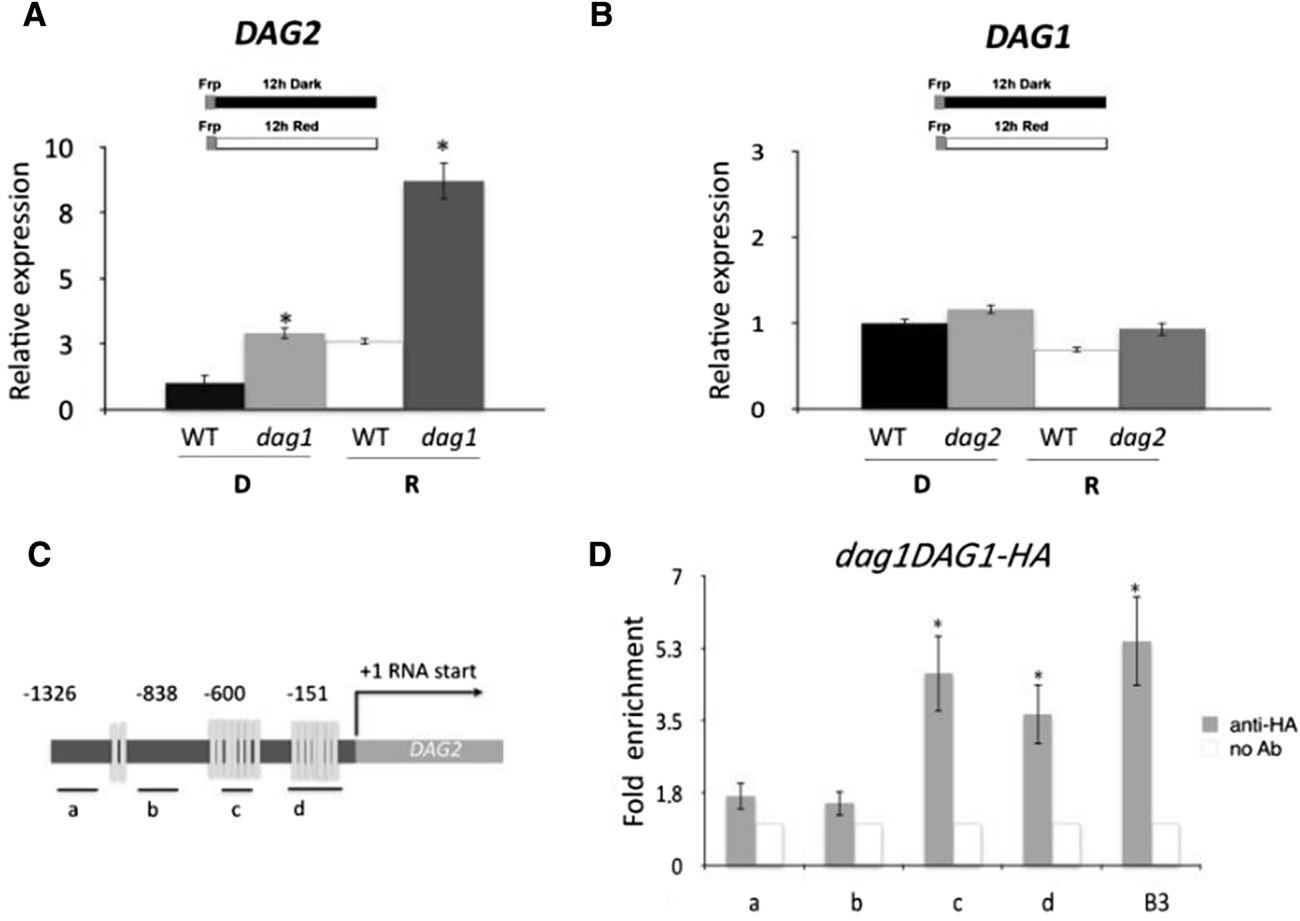
***DAG2*** **is directly regulated by DAG1.** Relative expression level of: *DAG2* in *dag1* mutant and wild-type (WT) seeds **(A)**, and of *DAG1* in *dag2* mutant and wild-type seeds **(B)**. Seeds were imbibed 12 hours in the dark (D), or under R light (R). Relative expression levels were normalized with that of the *UBQ10* gene and are presented by the ratio of the corresponding wild-type mRNA level in D, which was set to 1. Similar results were obtained from three independent experiments, and a typical result is presented with SD values. Significative differences were analyzed by *t*-test (*P ≤ 0,05). **(C)** Graphic representation of the *DAG2* promoter. Underlying thick lines marked by letters (a, b, c, d) are referred to different promoter fragments used for qPCR, containing 0 (a, b), 4 and 7 Dof sites respectively (c,d). **(D)** Chromatin from *dag1DAG1-HA* seeds was immunoprecipitated with anti-HA or without antibody, and the amount of DNA was measured by qPCR. B3 is referred to the positive control, fragment B3 of the *AtGA3ox1* promoter bound by DAG1-HA The values of fold enrichment are the average of three independent experiments presented with SD values. Significative fold enrichment was analyzed by *t*-test (*P ≤ 0,05).

We have previously investigated the genetic interactions between the *DAG2* and *DAG1* genes by isolating the *dag2dag1* double mutant, and showed that *DAG1* is epistatic over *DAG2* [8]. Since the function of DAG2 appears to be opposite to that of DAG1, we verified whether DAG1 and DAG2 would mutually affect their expression, by performing an RT- qPCR analysis in *dag1* and *dag2* mutant seeds imbibed for 12 hours in the dark or under R light. As shown in Figure 3A, expression of *DAG2* is significantly (approximately 3-fold) increased by lack of DAG1, irrespective of light conditions. Conversely, *DAG1* expression level in wild-type and *dag2* mutant seeds was comparable, both in the dark and under R light (Figure 3B).

To assess whether DAG1 regulates *DAG2* by directly binding to the *DAG2* promoter *in vivo*, we performed chromatin immunoprecipitation (ChIP) assays, utilizing the *dag1DAG1-HA* line previously reported [22,23]. A scheme of the *DAG2* promoter is reported in Figure 3C, showing the positions of the PCR fragments amplified for the ChIP assays, each containing different numbers of Dof binding sites: 0 (a, b), 4 (c) and 7 sites (d). Consistently, anti-HA antibodies revealed that the amplification of fragments c and d were the most efficient, compared to the positive control, the fragment B3 of the *AtGA3ox1* promoter bound by DAG1-HA, as previously reported [23]. On the contrary, the signal for fragments a and b was quite faint. No PCR product was present for any of the fragments in the sample precipitated without antibodies as a negative control, and not even for the negative control on wild-type seeds (Figure 3D; Additional file 1: Figure S1). These results indicate that DAG1 negatively regulates *DAG2* by directly binding the *DAG2* promoter.

### PIL5 negatively regulates DAG2 in the Dark

**Figure 4:**
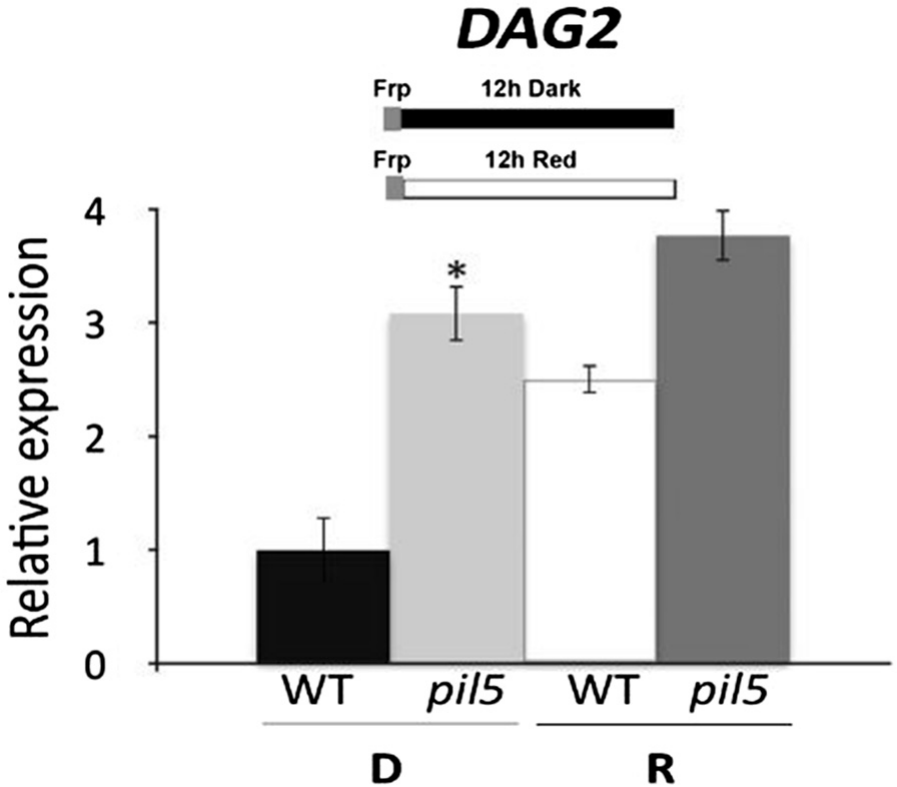
***DAG2*** **expression is repressed by PIL5.** Relative expression level of *DAG2* in *pil5* mutant and wild-type seeds. Seeds were imbibed 12 hours in the dark (D), or under R light (R). Relative expression levels were normalized with that of the *UBQ10* gene, and are presented by the ratio of the corresponding wild-type mRNA level in D, which was set to 1. Similar results were obtained from three independent experiments, and a typical result is presented with SD values. Significative differences were analyzed by *t*-test (*P ≤ 0,05).

Since DAG1 and DAG2 seem to have opposite roles in the phyB-mediated seed germination pathway, we wondered whether PIL5, which positively regulates *DAG1*, might negatively control the expression of *DAG2*. To verify this hypothesis, we analysed the expression of the *DAG2* gene in wild-type and *pil5* mutant seeds after 12 hours imbibition in the dark or under R light. Interestingly, as shown in Figure 4, the relative amount of *DAG2* in *pil5* mutant seeds in the dark was significantly higher than in the wild-type, suggesting that PIL5 negatively regulates the expression of *DAG2* in the dark. On the other hand, *DAG2* expression level in R light does not depend on PIL5, as it is degraded following interaction with phyB.

**Figure 5:**
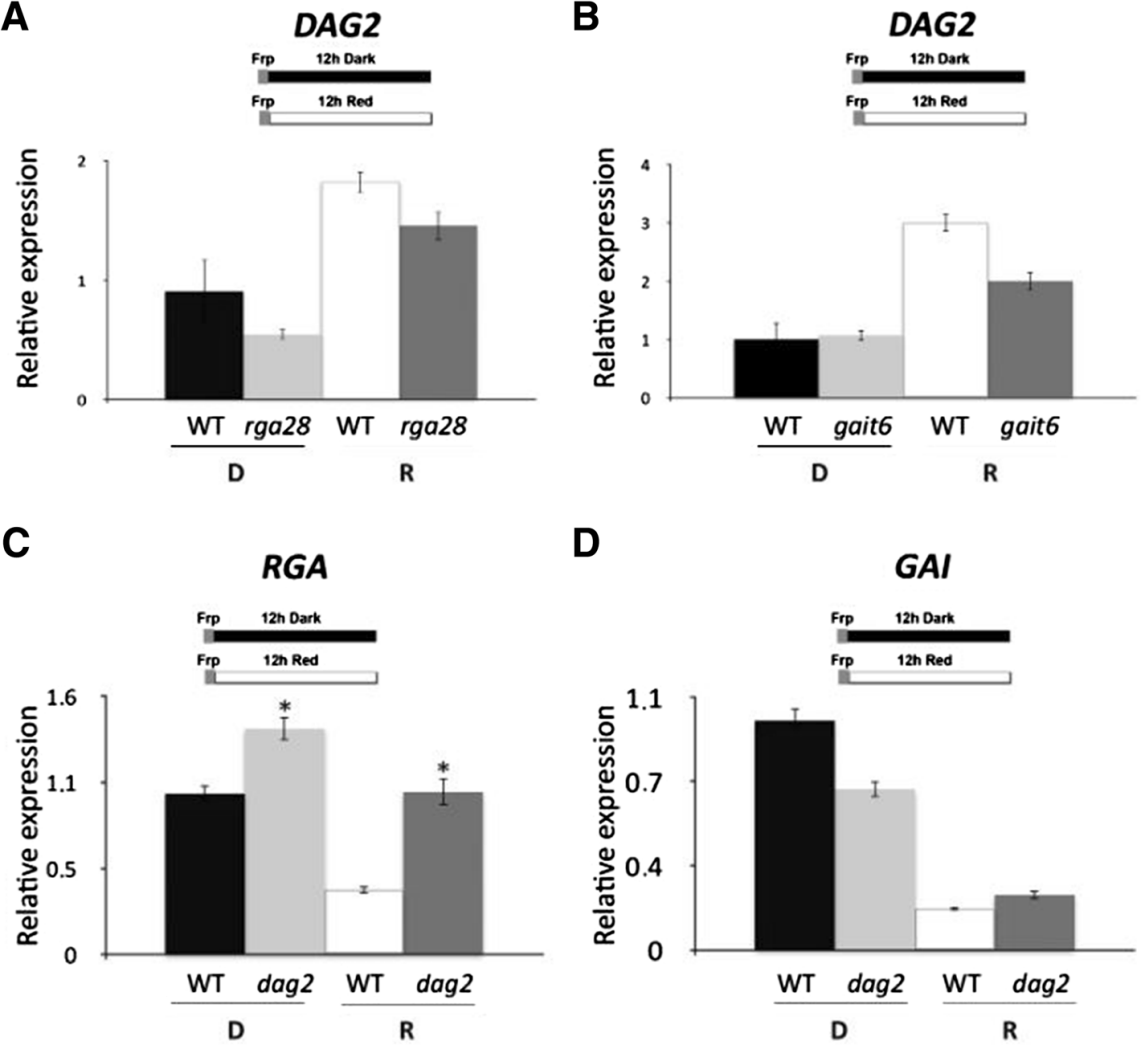
***DAG2*** **expression is regulated by RGA and GAI.** Relative expression level of: *DAG2* in *rga28* **(A)**, and *gai-t6* mutant seeds **(B)**, and of *RGA* **(C)** or *GAI* **(D)** in *dag2* mutant seeds, compared to wild-type seeds. Seeds were imbibed 12 hours in the dark (D), or under R light (R). Relative expression levels were normalized with that of *PP2A* (*At1g13320*) **(A, B)**, or of *UBQ10* **(C, D)**, and are presented by the ratio of the corresponding wild-type mRNA level in D, which was set to 1. Similar results were obtained from three independent experiments, and a typical result is presented with SD values. Significative differences were analyzed by *t*-test (*P ≤ 0,05).

The DELLA proteins GAI and RGA are negative regulators of seed germination, acting downstream of PIL5 [26]. In particular, we have recently shown that GAI and DAG1 mutually regulate their expression level and directly interact with each other [23]. Thus, we set to assess whether GAI and/or RGA might control the expression level of *DAG2*. Analysis of *DAG2* expression on *gai-t6* and *rga28* mutant seeds and on the corresponding Col-0 wild-type seeds, imbibed 12 hours in the dark or under R light, revealed that neither GAI nor RGA control *DAG2* expression, as the relative amount of *DAG2* mRNA was similar in the *gai-t6* and *rga28* single mutants compared to the wild-type, under both light conditions (Figure 5A, B). To verify whether DAG2 might regulate expression of these DELLA proteins, we analysed the expression of *GAI* and *RGA* in *dag2* mutant seeds compared to the wild-type. As shown in Figure 5C, the expression of the *RGA* gene was significantly increased in *dag2* mutant seeds, whereas *GAI* expression in wild-type and *dag2* mutant seeds was not significantly different, both in the dark and under R light (Figure 5D).

These results point to DAG2 as a positive component of light-mediated signalling pathway, downstream of PIL5 and in turn controlling the DELLA protein RGA in the phyB signalling pathway.

We then verified whether expression of some factors known to be involved in the phyA-signalling pathway [27] may be affected in *dag2* mutant seeds. In particular, we analysed expression of the FR light-regulated *ARABIDOPSIS THALIANA HOMEOBOX PROTEIN 2* (*ATHB2)* and *PHYTOCHROME INTERACTING FACTOR 3-LIKE 2* (*PIL2*) genes, of the GA-regulated *GA-STIMULATED ARABIDOPSIS 4* and *6* (*GASA4* and *GASA6*), and of the ABA signalling gene *ABA INSENSITIVE 4* (*ABI4)*. Our data revealed that under phyA-dependent conditions neither expression of *ATHB2* and *PIL2*, nor that of *ABI4* were affected in the *dag2* mutant (Additional file 2: Figure S2) whereas expression of both *GASA4* and *GASA6* was downregulated, thus opening the possibility that DAG2 may also play a role in phyA signalling.

### The *dag2* mutation alters GA metabolism

It has been shown that phyB controls the ratio of GA and ABA levels during seed germination by altering the expression of different GA and ABA metabolic genes through PIL5 [18,26]. In particular, DAG1 directly represses the GA biosynthetic gene *AtGA3ox1* in cooperation with GAI [22,23], and its inactivation affects expression of the ABA metabolic genes *ABA1, ABA2* and *CYP707A2* [22].

**Figure 6:**
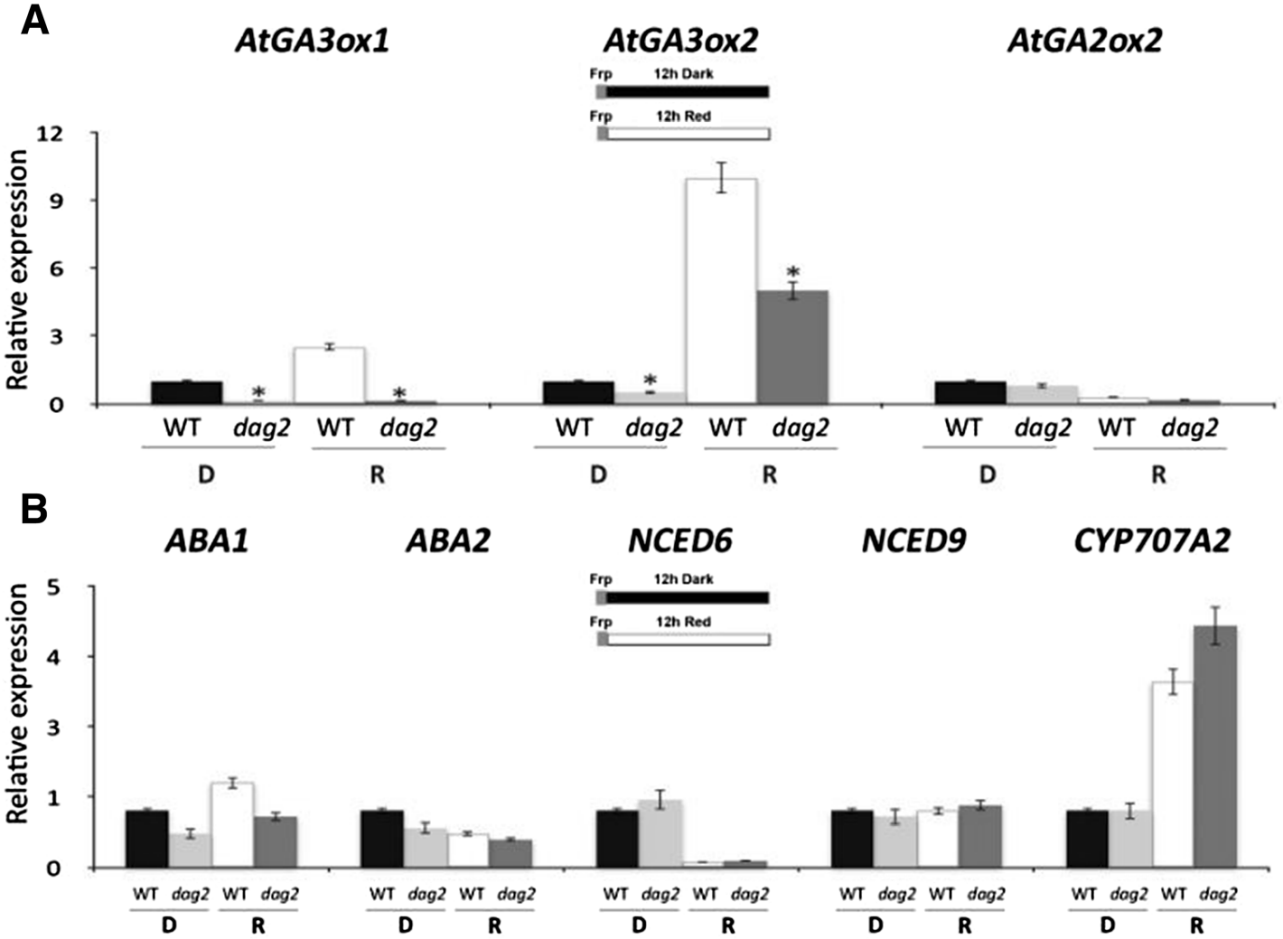
**Mutation of the** ***DAG2*** **gene affects GA biosynthesis.** Relative expression level of *AtGA3ox1*, *AtGA3ox2* and *AtGA2ox2* **(A)**, and of *ABA1, ABA2, NCED6, NCED9* and *CYP707A2* **(B)** in *dag2* mutant seeds compared to wild-type seeds. Seeds were imbibed 12 hours in the dark (D), or under R light (R). Relative expression levels were normalized with that of the *UBQ10* gene, and are presented by the ratio of the corresponding wild-type mRNA level in D, which was set to 1. Similar results were obtained from three independent experiments, and a typical result is presented with SD values. Significative differences were analyzed by *t*-test (*P ≤ 0,05).

As DAG2 seems to have a role opposite to DAG1 in seed germination, we investigated whether DAG2 would regulate the expression of GA and ABA metabolic genes in germinating seeds. We performed a RT-qPCR analysis of the expression of the GA biosynthetic genes *AtGA3ox1, AtGA3ox2* and of the catabolic gene *AtGA2ox2* in *dag2* and wild-type seeds imbibed 12 hours in the dark or under R light. As shown in Figure 6A, the expression of both GA biosynthetic genes was significantly reduced in *dag2* mutant seeds irrespective of light conditions, whereas the catabolic gene *AtGA2ox2* was expressed similarly in *dag2* and wild-type seeds (Figure 6A).

As for ABA metabolism, we analysed the expression level of the biosynthetic genes *ABA1*, *ABA2, NCED6* and *NCED9,* and of the catabolic gene *CYP707A2*, on *dag2* and wild-type seeds imbibed 12 hours in the dark or under R light. The expression profile of the biosynthetic genes, as well as of the catabolic gene *CYP707A2* did not show significant differences in *dag2* and wild-type seeds (Figure 6B).

**Figure 7:**
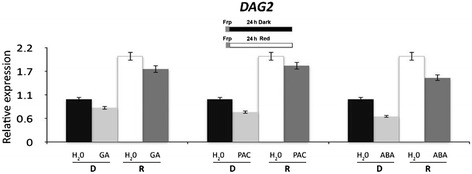
***DAG2*** **expression is not altered by ABA or GA.** Relative expression level of *DAG2* in wild-type seeds imbibed 24 hours in the presence of GA, of Paclobutrazol, an inhibitor of GA biosynthesis, or of ABA in the dark (D), or under R light (R), compared to seeds imbibed in water as a control (H_2_O). Relative expression levels were normalized with that of the *UBQ10* gene, and are presented by the ratio of the corresponding mRNA level in seeds imbibed in water, which was set to 1. Similar results were obtained from three independent experiments, and a typical result is presented with SD values.

We have previously shown that the sensitivity of seeds to GA is affected by mutation of the *DAG2* gene: a concentration of GA 10-fold higher than for wild-type seeds was needed for *dag2* mutant seeds to attain 50% germination [8]. To verify whether GA affect *DAG2* expression, we carried out an RT-qPCR analysis on wild-type seeds imbibed 24 hours in the presence of GA or of paclobutrazol, an inhibitor of GA biosynthesis. Since GA metabolism is controlled by the ABA level [18], we also checked *DAG2* expression on wild-type seeds imbibed 24 hours in the presence of ABA. The results of this analysis did not show any significant difference in *DAG2* transcript levels in all conditions tested, clearly showing that the *DAG2* gene is not regulated by GA nor by ABA irrespective of light conditions (Figure 7).

### Inactivation of the *DAG2* gene does not affect embryo development

**Figure 8:**
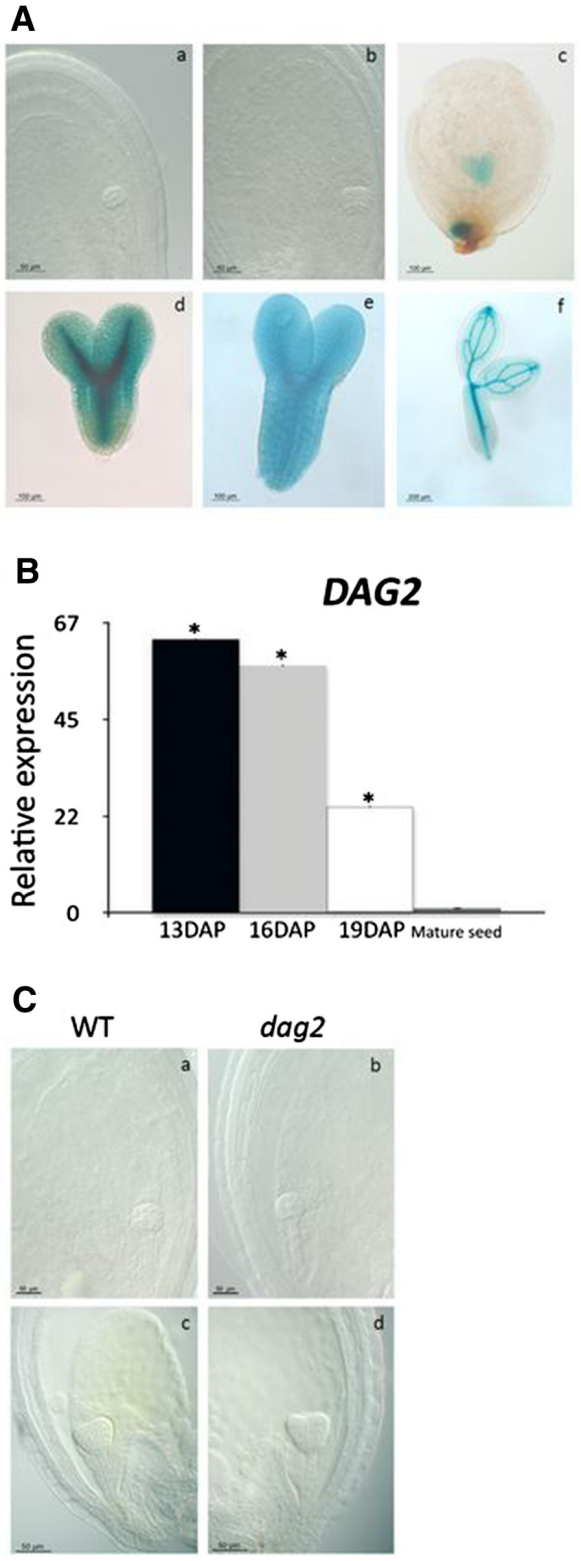
***DAG2*** **inactivation does not affect embryo development.** Histochemical staining of *DAG2:GUS* during embryogenesis, in early globular, globular, heart, late heart, torpedo and mature embryo **(A)**.Relative expression level of *DAG2* in wild-type seeds at 13, 16 and 19 Days After Pollination (DAP), and in mature seeds (28 DAR). Relative expression levels were normalized with that of the *UBQ10* gene, and are presented by the ratio of the corresponding mRNA level in mature seeds, which was set to 1. Similar results were obtained from three independent experiments, and a typical result is presented with SD values. Significative differences were analyzed by *t*-test (*P ≤ 0,05) **(B)**. Phenotypes of wild-type (a, c) and *dag2* mutant (b, d) embryos, at globular (a, b) and heart stage (c, d) **(C)**.

We have recently shown that *DAG1* is expressed during embryo development, and that lack of DAG1 affects this process [24]. Thus, we set to assess whether also DAG2 is required for embryo development. We first analyzed the expression of *DAG2* during embryo development by histochemical GUS analysis of seeds of the *DAG2:GUS* transgenic line. GUS activity was observed in embryos at the heart, torpedo, and bent-cotyledon stages. Interestingly, GUS staining was extended to all cells at the heart stage, whereas from the torpedo stage on it was restricted to the procambium (Figure 8A). These results were confirmed and extended to later seed development stages by a RT-qPCR analysis on wild-type embryos at 13, 16 and 19 Days After Pollination (DAP), compared to mature seeds, to verify whether the *DAG2* gene was expressed also during seed maturation.

Expression of *DAG2*, at 13 and 16 DAP was extremely high (63- and 57-fold the basal level, respectively), and gradually decreased at 19 DAP (24-fold) compared to mature seeds (Figure 8B).

Despite the high expression level of the *DAG2* gene during embryo and seed development, microscopic analysis of *dag2* mutant embryos did not reveal any noticeable phenotypical alteration (Figure 8C).

## Discussion

We had previously shown that the *dag2* mutant has seeds which require higher light fluences and higher GA levels than wild-type ones to germinate [8], suggesting a positive role of the Dof transcription factor DAG2 in the regulation of seed germination.

Here, we have expanded our analysis of the function of DAG2 and we confirm the positive role of DAG2 in seed germination and provide molecular and genetic evidences that assign this protein to the phyB/PIL5 pathway.

To date the molecular pathway controlling seed germination has been partially elucidated. In this model PIL5 acts as the master repressor, which inhibits seed germination in the dark partly by activating the expression of the genes encoding the DELLA proteins RGA and GAI - which repress germination acting as negative GA signaling components - and of the transcription factors *ABA INSENSITIVE 3* and *5* (*ABI3* and *ABI5*) - which function as positive ABA signaling molecules [26,28]. Other transcription factors acting as repressors have been added in this pathway: the bHLH transcription factor SPATULA (SPT) [29], the C3H-type zinc finger protein SOMNUS (SOM) [30], and the Dof transcription factor DAG1, which we have shown to directly regulate the GA biosynthetic gene *AtGA3ox1*, with the cooperation of GAI [22,23].

DAG1 and DAG2 share 77% overall aminoacidic identity, with 100% identity in the Dof domain and, based on the opposite germination properties of *dag1* and *dag2* mutant seeds, we had assumed that the function of these two Dof proteins was opposite. This was also supported by *DAG1* overexpression, which caused phenotypes similar to mutation of *DAG2* [8]. Consistently, germination of *dag2* mutant seeds in phyB-dependent conditions (i.e. under R light) was significantly reduced compared to wild-type seeds, whereas *dag1* seeds showed a higher germination frequency [21,22]. In addition, *DAG2* expression is induced by exposure to R light, as opposed to *DAG1*, whose transcript level is lower in R light than in the dark [22].

Analysis of the germination properties of *dag2dag1* double mutant seeds revealed that the *dag1* mutation is epistatic over the *dag2* one [8]. Consistent with these previous reports, here we showed that *DAG2* expression is negatively controlled by DAG1, and that DAG1 directly binds the *DAG2* promoter as demonstrated by ChIP assay.

This provides molecular support to the genetic evidence of the epistatic relationship between these two Dof proteins shown in previous work [8]. We show here that *DAG2* is also repressed by PIL5, since the *DAG2* mRNA level is significantly increased in *pil5* mutant seeds in the dark but not under R light, where PIL5 is degraded following interaction with phyB in its activated form (Pfr).

Since DAG1 directly interacts with GAI, and cooperates with this DELLA protein in repressing the GA biosynthetic gene *AtGA3ox1* [23], and in the light of the opposite role of DAG2 in this molecular pathway, one could hypotesize a relationship of DAG2 with RGA or GAI. Interestingly, our results revealed that expression of *RGA*, but not of *GAI*, is significantly affected in *dag2* mutant seeds exposed to R light, suggesting that DAG2 may negatively regulate this *DELLA* gene, whereas expression of *DAG2* is not likely to be controlled by both RGA and GAI, as *DAG2* transcript levels are similar in *rga28* and in *gai-t6* mutant seeds compared to wild-type seeds, in both light conditions.

Our expression analysis under phyA-dependent conditions further supports the notion that DAG2 acts in the phytochrome-mediated seed germination. In fact, of the marker genes of the phyA-dependent germination pathway we analyzed, *PIL2*, *ATHB2*, and *ABI4* remained unaffected in the *dag2* mutant, while *GASA4* and *GASA6* were severely downregulated - consistent with the role of DAG2 in the positive control of GA biosynthesis - opening the interesting possibility that DAG2 participates also in phyA signalling.

It should be noted that GASA4 has been previously characterised as a regulatory protein, induced by GA and involved in seed development and germination, independently of light conditions [31,32].

Phytochromes promote seed germination partly through GA. Red light induces the expression of the two GA anabolic genes *GA3-oxidase* genes *GA3ox1* and *GA3ox2*, whereas it represses the GA catabolic gene *GA2ox2* [33,34]. Consistent with a positive role of DAG2 in seed germination, mutation of the *DAG2* gene severely affects expression of both *AtGA3ox1* and *AtGA3ox2*, although it does not alter the expression level of *AtGA2ox2*. Unlike DAG1, DAG2 does not seem to play its function through regulation of ABA metabolism, as the expression profile of the ABA metabolic genes tested is quite similar in *dag2* and wild-type seeds [22].

In recent years, the molecular mechanisms underlying light-mediated seed germination has been partly elucidated; however, it still remains an open question which are the positive regulators of this process. In fact, so far only LONG HYPOCOTYL IN FAR RED1 (HFR1) has been identified as a positive regulator of seed germination: HFR1 acts upstream of PIL5 and interacts directly with PIL5 thus sequestering it to prevent it from binding to its target genes [35]. Interestingly, germination of *hfr1* mutant seeds under phyB-dependent germination conditions is very similar to that of *dag2* mutant seeds, strengthening the notion that DAG2 is also a positive regulator in the phyB-dependent seed germination pathway.

As previously reported, *DAG2* and *DAG1* show a very similar expression profile, restricted to the vascular tissue [8], and we showed that during embryo development, *DAG1* is expressed from late globular stage [22,24]. We also showed that *dag1* mutant embryos displayed abnormal cell divisions at globular stage, altering the radial symmetry of the embryo axis [24].

Here we showed that, in contrast with DAG1, although also *DAG2* is expressed during embryo development, its absence does not produce obvious embryo phenotypes.

## Conclusions

Our genetic and molecular data indicate that DAG2 is a new positive factor of the phyB/PIL5-mediated seed germination pathway. DAG2 is located downstream PIL5 and DAG1, which directly represses *DAG2* expression. Consistent with previous genetic data, DAG2 plays an opposite role to DAG1, although our results indicate that DAG2 acts on GA, but not on ABA, metabolism.

## Methods

### Plant material and growth conditions

*dag2* is the allele described in Gualberti *et al.* [8] in Ws-4 ecotype.

All *Arabidopsis thaliana* lines used in this work were grown in a growth chamber at 24/21°C with 16/8-h day/night cycles and light intensity of 300 μmol/m^−2^ s^−1^ as previously described [7,22].

### Seed germination assays

All seeds used for germination tests were harvested from mature plants grown at the same time, in the same conditions, and stored for the same time (28 Days After Ripening, DAR) under the same conditions. Germination assays were performed according to Gabriele *et al*. [22]. For phyB-dependent germination experiments, seeds were exposed to a pulse of FR light (40 μmol m^−2^ s^−1^), then a pulse of R light (90 μmol m^−2^ s^−1^) and subsequently kept in the dark for 5 days. Germination assays were repeated with three seed batches, and one representative experiment is shown. Bars represent the mean ± SEM of three biological repeats (25 seeds per biological repeat). P values were obtained from a Student’s unpaired two-tail *t* test comparing the mutant with its control (* = p ≤ 0,05).

### Expression analysis

For expression analysis, seeds were imbibed for 12 or 24 hours, on five layers of filter paper, soaked with 5 ml water, exposed to a pulse of FR (40 μmol m^−2^ s^−1^), then incubated in the dark or under R light (90 μmol m^−2^ s^−1^), in the presence of PAC (100 μM) to prevent de-novo GA biosynthesis in response to light [26]. For phyA-dependent conditions, seeds were treated according to Oh *et al*., 2006 [25]. RNA extraction and RT-qPCR were performed according to Gabriele *et al*. [22]. Quantification of gene expression was expressed in comparison to the reference gene (See legends of figures), and relative expression ratio was calculated based on the qRT-PCR efficiency (E) for each gene and the crossing point (CP) deviation of our target genes versus a control [36]. The expression analyses were repeated in comparison with a second reference gene (Additional file 3: Figure S3).

Three independent biological replicates were performed, and one representative experiment is reported. Significative differences were analyzed by *t*-test (*P ≤ 0,05). The primers used for the assays are listed in Additional file 4: Table S1.

### ChIP analysis

The *dag1DAG1-HA* line is the one previously described in Gabriele *et al.* [22]. ChIP was performed as previously described [22], with 12 hours imbibed seeds. Antibodies against HA tag (Santa Cruz, CA, USA) were used for immunoprecipitation. Equal amounts of starting material and ChIP products were used for qPCR reaction. The primers used are listed in Table S1. Three independent biological replicates were performed. Significative differences were analyzed by *t*-test (*P ≤ 0,05).

### Microscopy and GUS analysis

Analysis of *dag2* and wild-type embryos was performed under an Axioskop 2 plus microscope (Zeiss).

The *DAG2:GUS* line is the one described in Gualberti *et al.* [8]. Histochemical staining and microscopic analysis were carried out according to Blazquez *et al.* [37]. Stained embryos (after washing in 70% ethanol) were analysed and photographed under an Axioskop 2 plus microscope (Zeiss).

### Availability of supporting data

All the supporting data of this article are included as additional files (Additional files 1, 2 and 3: Figures S1-S3; Additional file 4: Table S1).

## Additional files

Additional file 1:
**ChIP analysis of wild-type (WS) seeds immunoprecipitated with anti-HA antibody or without antibody.**
Additional file 2:
**Relative expression levels of** ***ATHB2, PIL2, GASA4, GASA6*** **and** ***ABI4*** **in wild-type (WT) and** ***dag2*** **mutant seeds.** Relative expression levels were normalized with that of the *UBQ10* gene. Additional file 3:
**Expression analysis with a second reference gene.** Relative expression levels of *DAG2* in wild-type seeds in the dark (D), Red (R) (A), or Far Red (FR) (B) light, in dry seeds (0h), or imbibed 12 (12h), 24 hours (24h) in the dark, under White (W) or Red light (C), normalized with the *PP2A* gene. Relative expression levels of *DAG2* in *dag1* (D), *pil5* (F), *rga28* (G), *gai-t6* (H) seeds compared to WT. Relative expression levels of *DAG1* (E), *RGA* (I), GAI (L), in *dag2* seeds compared to WT. The expression levels were normalized with *PP2A* (D, F, E, I, L), or with *eIF1α* (*At5g60390)* (G, H). Relative expression levels of GA (M) or ABA (N) metabolic genes in *dag2* seeds compared to WT, normalized with *PP2A* gene. Relative expression levels of *DAG2* in WT seeds imbibed in the presence of ABA, or GA, or PAC (O), in WT embryos at 13, 16, 19 DAP (P), normalized with *PP2A* gene. Relative expression levels of *ATHB2, PIL2, GASA4, GASA6* and *ABI4* in *dag2* seeds compared to WT (Q), normalized with *PP2A*. Additional file 4: Table S1: List of the primers used for expression analyses and for the ChIP assays.

## Abbreviations

DOF: DNA Binding With One Finger
DAG1: Dof AFFECTING GERMINATION 1
DAG2: Dof AFFECTING GERMINATION 2
phyB: Phytochrome B
PIL5: PHYTOCHROME INTERACTING FACTOR3-LIKE 5
GAI: GA INSENSITIVE
RGA: REPRESSOR OF *ga1-3*
ABI3: ABA INSENSITIVE 3
ABI5: ABA INSENSITIVE 5
SPT: SPATULA
SOM: SOMNUS
HFR1: LONG HYPOCOTYL IN FAR RED1
ATHB2: ARABIDOPSIS THALIANA HOMEOBOX PROTEIN 2
GASA4: 6, GA-STIMULATED ARABIDOPSIS 4, 6
PIL2: PHYTOCHROME INTERACTING FACTOR3-LIKE 2
ABI4: ABA Insensitive 4,ABA, Abscissic Acid
GA: Gibberellins
PAC: Paclobutrazol
ChIP: Chromatin Immuno Precipitation
RT-qPCR: quantitative reverse transcriptase-polymerase chain reaction
W light: White light
R light: Red light
D: Dark
FR Light: Far Red Light
DAP: Days After Pollination
DAR: Days After Ripening
GUS: β-glucuronidase
HA: Heme Agglutinin
