# Supplementary figures and images for: DOF AFFECTING GERMINATION 2 is a positive regulator of light-mediated seed germination and is repressed by DOF AFFECTING GERMINATION 1
Source: BMC Plant Biol. 2015 Mar 4;15:72. doi: 10.1186/s12870-015-0453-1 (PMC4355143; doi:10.1186/s12870-015-0453-1)

*WS*

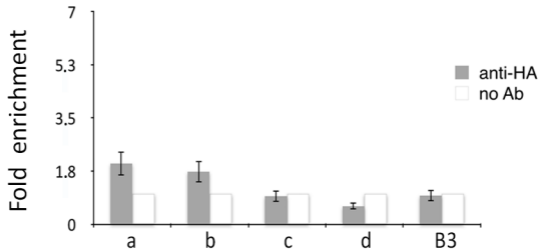

Supplement: Additional file 1: — ChIP analysis of wild-type (WS) seeds immunoprecipitated with anti-HA antibody or without antibody. [file 12870_2015_453_MOESM1_ESM.pdf]

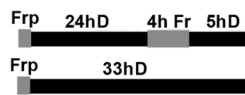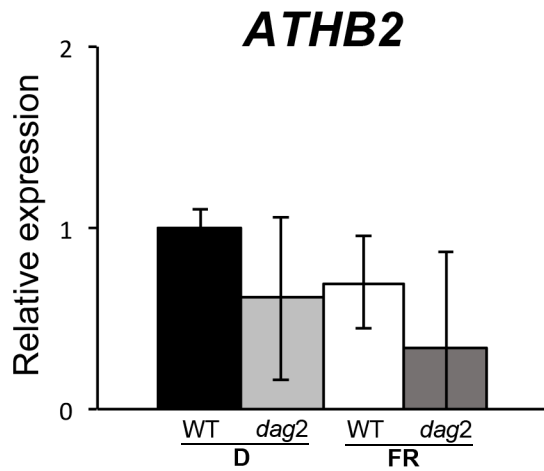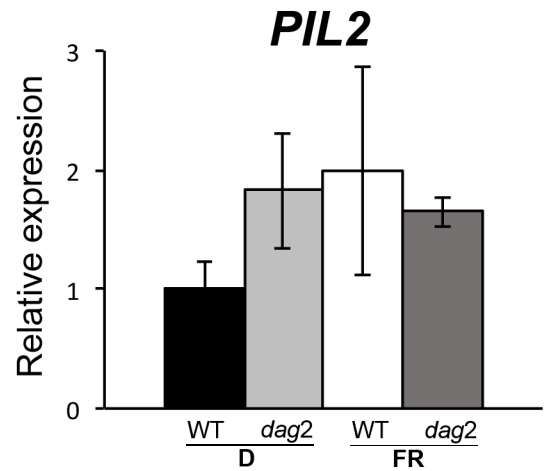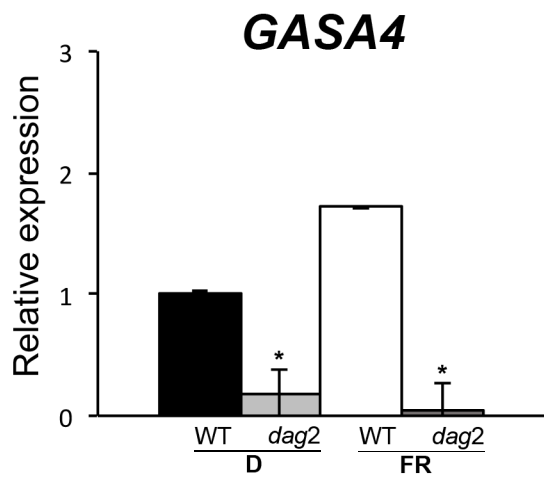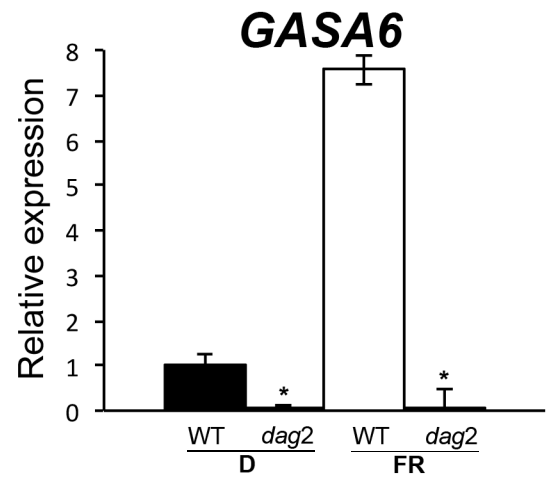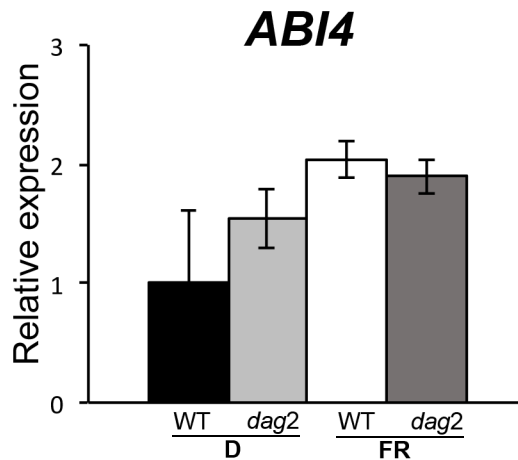

Supplement: Additional file 2: — Relative expression levels of ATHB2, PIL2, GASA4, GASA6 and ABI4 in wild-type (WT) and dag2 mutant seeds. Relative expression levels were normalized with that of the UBQ10 gene. [file 12870_2015_453_MOESM2_ESM.pdf]
