# Supplementary material for: DOF AFFECTING GERMINATION 2 is a positive regulator of light-mediated seed germination and is repressed by DOF AFFECTING GERMINATION 1
Source: BMC Plant Biol. 2015 Mar 4;15:72. doi: 10.1186/s12870-015-0453-1 (PMC4355143; doi:10.1186/s12870-015-0453-1)
Supplement: Additional file 4: Table S1: — List of the primers used for expression analyses and for the ChIP assays. [file 12870_2015_453_MOESM4_ESM.pdf]

Specific primer sets used in the ChIP-qPCR:

	Forward	Reverse
pGA3ox1	AGA CAA CAT TGG CCC TTT CT	ATT GTG AAA GGG GGA GAC AT
pDAG2-A	CCA TTA CAA CAT GTT AGT TTG ATA GC	AAG ACT GTT CAG CTA TTA ATT GTT TTG
pDAG2-B	TGT AGC TAG GCG ATT TGA TGG	CTA TTT CTC GGC CTC CCA TT
pDAG2-C	CGG TCA CGA GAC ATC ATC AC	CAT CCG TCT AAT TCG GGA GA
pDAG2-D	AAG GCA ACG CAT AGA AGA GC	CCG ACA AAG AGA AAG CCA GA

Specific primer sets used in the qRT-PCR:

	Forward	Reverse
ABA1	GAT GCA GCC AAA TAT GGG TCA AGG	GCC ATT GCA TGG ATA ATA GCG ACT C
ABA2	AGA GGT GTT TGC ATG ATT CCT GAG C	TCC AGT GAT CAA TGC CAC TTT ACC C
NCED9	AAC CGC CGC TAT GGT TTT AGA CG	CCA GTC ACC GGA AGG TTA TGC AC
NCED6	ACC GGG TCG GAT ATA AAT TGG GTT G	CCC GGG TTG GTT CTC CTG ATT C
CYP707A2	ATG GGG TTG CCT TAC ATC GGA GA	TGG CTT GAA CAA GTG AGC TTT GCT
GA3ox1	GCT TAA GTC TGC TCG GTC GG	AGT GCG ATA CGA GCG ACG
GA3ox2	ACG TCG GTG ACT TGC TCC A	GTT AAC CCT GGC TCG GTG AA
GA2ox2	TCC GAC CCG AAC TCA TGA CT	CGG CCC GGT TTT TAA GAG AC
DAG1	TTG TCG AAG GTA TTG GAC CGA	CCG ACT GGG ACG TTA CGA AG
RGA	CAT TCC CGG AAA CGC GAT TTA TCA G	TCA CCG TCG TTC CTA TGA CTC CA
GAI	AGC GTC ATG AAA CGT TGA GTC AGT G	TGC CAA CCC AAC ATG AGA CAG C
PP2A	TGC TGA AGA GTT GGT CCT G	GAA GCG ATA CTG CAC GAA GA
GASA4	ATG TGA AGT GGA GCC AGA AAC G	GCA AGC CTT GTG GTA CTG TGT C
GASA6	AAA TGC CTT TGT GTC CCT CCA G	GGT CCA CCT TGT TGA GTC TTC C
PIL2	AAC CCG TGA GCA GAT GAA GTA AAC	ATG AAG AAG AAG ACC GTG AAT CCG
ATHB2	TGC GAG TTC TTA CGG AGA TG	AGT AGT GGG TGG GCT CAT GT
eIF1α	TGA GCA CGC TCT TCT TGC TTT CA	GGT GGT GGC ATC CAT CTT GTT ACA
ABI4	CAG ATG GGA CAA TTC CAA CA	CTC CTT GTT CCT GCC CTA AC
ACT	GACCAGCTCTTCCATCGAGAA	CAAACGAGGGCTGGAACAAG
DAG2	TTG GGG ATC AAA TGA AGG AG	TTC CAT GAC TCT TCT GTT GTT GA
UB10	GGC CTT GTA TAA TCC TGA TGA ATA AG	AAA GAG ATA ACA GAC GGA AAC ATA GT
