# Supplementary material for: DOF AFFECTING GERMINATION 2 is a positive regulator of light-mediated seed germination and is repressed by DOF AFFECTING GERMINATION 1
Source: BMC Plant Biol. 2015 Mar 4;15:72. doi: 10.1186/s12870-015-0453-1 (PMC4355143; doi:10.1186/s12870-015-0453-1)
Supplement: Additional file 3: — Expression analysis with a second reference gene. Relative expression levels of DAG2 in wild-type seeds in the dark (D), Red (R) (A), or Far Red (FR) (B) light, in dry seeds (0h), or imbibed 12 (12h), 24 hours (24h) in the dark, under White (W) or Red light (C), normalized with the PP2A gene. Relative expression levels of DAG2 in dag1 (D), pil5 (F), rga28 (G), gai-t6 (H) seeds compared to WT. Relative expression levels of DAG1 (E), RGA (I), GAI (L), in dag2 seeds compared to WT. The expression levels were normalized with PP2A (D, F, E, I, L), or with eIF1α (At5g60390) (G, H). Relative expression levels of GA (M) or ABA (N) metabolic genes in dag2 seeds compared to WT, normalized with PP2A gene. Relative expression levels of DAG2 in WT seeds imbibed in the presence of ABA, or GA, or PAC (O), in WT embryos at 13, 16, 19 DAP (P), normalized with PP2A gene. Relative expression levels of ATHB2, PIL2, GASA4, GASA6 and ABI4 in dag2 seeds compared to WT (Q), normalized with PP2A. [file 12870_2015_453_MOESM3_ESM.pdf]

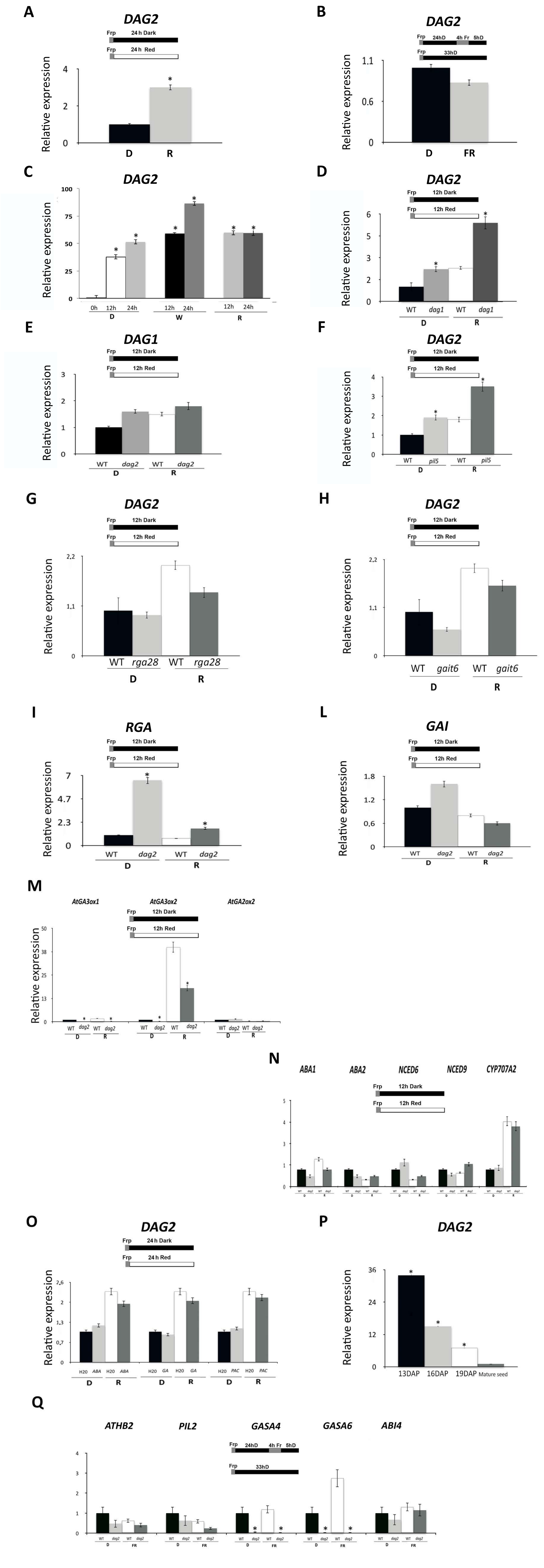
A
DAG2
Frp 24 h Dark
Frp 24 h Red
Relative expression
D
R
B
DAG2
Frp 24hD 4h Fr 5hD
Frp 33hD
Relative expression
D
FR
C
DAG2
Relative expression
0h 12h 24h
D
12h 24h
W
12h 24h
R
D
DAG2
Frp 12h Dark
Frp 12h Red
Relative expression
WT dag1 WT dag1
D R
E
DAG1
Frp 12h Dark
Frp 12h Red
Relative expression
WT dag2 WT dag2
D R
F
DAG2
Frp 12h Dark
Frp 12h Red
Relative expression
WT pil5 WT pil5
D R
G
DAG2
Frp 12h Dark
Frp 12h Red
Relative expression
WT rga28 WT rga28
D R
H
DAG2
Frp 12h Dark
Frp 12h Red
Relative expression
WT gait6 WT gait6
D R
I
RGA
Frp 12h Dark
Frp 12h Red
Relative expression
WT dag2 WT dag2
D R
L
GAI
Frp 12h Dark
Frp 12h Red
Relative expression
WT dag2 WT dag2
D R
M
AtGA3ox1
AtGA3ox2
AtGA2ox2
Frp 12h Dark
Frp 12h Red
Relative expression
WT dag2 WT dag2
D R
N
ABA1
ABA2
NCED6
NCED9
CYP707A2
Frp 12h Dark
Frp 12h Red
O
DAG2
Frp 24 h Dark
Frp 24 h Red
Relative expression
H20 ABA H20 ABA
H20 GA H20 GA
H20 PAC H20 PAC
D R
P
DAG2
Relative expression
13DAP 16DAP 19DAP Mature seed
Q
ATHB2
PIL2
GASA4
GASA6
ABI4
Frp 24hD 4h Fr 5hD
Frp 33hD
WT dag2 WT dag2
D FR
